# How context influences the processing of relevant information and judgment accuracy—the role of information restriction in judgment processes in diagnosing misconceptions

**DOI:** 10.3389/fpsyg.2024.1405756

**Published:** 2024-09-25

**Authors:** Andreas Rieu, Katharina Loibl, Timo Leuders

**Affiliations:** ^1^Institute for Mathematics Education, University of Education Freiburg, Freiburg, Germany; ^2^Institute of Psychology, University of Education Freiburg, Freiburg, Germany

**Keywords:** diagnostic judgment, restriction of information, cognitive bias, judgment processes, teacher training

## Abstract

To adapt teaching to the prerequisites of students, teachers have various options at their disposal to gather and process information as the basis to form a judgment, such as carrying out tests, talking to and observing the behavior of students, or administering tasks. The complexity of such a judgment arises from the multitude of observations and their different possible explanations. This complexity might be reduced when teachers focus on one hypothesis instead of considering multiple hypotheses, interpret information in a confirmatory way, and not collect diagnostically relevant information. However, in this way, they run the risk of undesirable biased judgments. It therefore seems important to improve diagnostic judgments by selecting and processing information in a more reflective way. Research indicates that if information on a student is not easily available but restricted (e.g., by time pressure, difficult access to the student or high effort), a teacher who wants to make a careful decision is forced to rely on more reflective processes in the selection of tasks and in the interpretation of solutions. The present experimental study therefore investigates how the restricted availability of information in a specific diagnostic situation—when diagnostically inexperienced prospective mathematics teachers determine misconceptions in decimal fractions—influences the underlying cognitive processes. We assume that restricting the availability of information on student behavior augments the attentional focus and therefore reduces cognitive biases. Such more reflective processing can be observed by an increased time spent per piece of information, which should lead to the processing of relevant information and further increase judgment accuracy. To investigate these hypotheses, prospective teachers without prior knowledge in diagnosing misconceptions (*N* = 81) were asked to diagnose misconceptions on decimal fractions of virtual students by collecting information on students’ solutions. Data concerning the effects of restricting the availability of information on teachers’ cognitive processes were analyzed. The results show that with restricted information, participants indeed select a greater proportion of diagnostically relevant tasks, which positively influences judgment accuracy. These results are discussed with respect to their significance for framing teacher training and for further research.

## 1 Introduction

A relevant factor for successful teaching is the adaptive consideration of students’ learning prerequisites, which in turn demands a sufficiently high accuracy of the teachers’ judgments of their students’ dispositions. While this judgment accuracy, often measured by some type of correlation between the judgment and an external criterion, is an expression of the judgment *quality*, it says little about information processing occurring during the formation of a judgment. Investigating the effects of cognitive processes and influencing factors on the judgment process therefore requires cognitive modeling and experimental investigations (Loibl et al., 2020). Several recent studies address information processing during the genesis of judgments and generate evidence that the processes taking place are influenced by personal dispositions such as knowledge and situational characteristics such as stress or time pressure (Becker et al., 2023; Rieu et al., 2022).

However, human judgment processes seem to be cognitively demanding, and the underlying information processing does not always proceed in a reflective way: cognitive biases such as the Pygmalion effect (Rosenthal and Jacobson, 1968) or the Halo effect (Lance et al., 1994) can be the cause of a subjectively distorted perception of the judgment situation and overly selective processing of information, which likely results in erroneous, so-called biased judgments (Rieu et al., 2024). These biases have been observed in complex judgment situations and, for example, occur as processing of information that confirms a certain assumption and as avoiding information that contradicts this assumption (confirmation or avoidance bias, cf. Ehrlinger et al., 2016; Wilke and Mata, 2012). A typical judgment situation threatened by such biases is when mathematics teachers are diagnosing students with respect to their knowledge or misconceptions about decimal fractions: An incorrectly solved task cannot be unequivocally attributed to a single misconception; instead, an accurate judgment can be made only by considering the responses to several tasks (e.g., Stacey, 2005). Therefore, teachers need to recognize the ambiguity of the situation and then specify their judgment with additional information (Rieu et al., 2024). They therefore purposefully select tasks that can be expected to deliver information and reduce ambiguity, so-called diagnostically relevant tasks (Kron et al., 2021). Each step toward the final judgment represents a demanding cognitive process; since the situation must be correctly perceived, hypotheses must be generated, and depending on these hypotheses, the tasks and their expected outcomes must be evaluated (Herppich et al., 2017).

To reduce the cognitive complexity of information processing, teachers may tend to simplify these processes (1) by not recognizing the ambiguity of the situation and thus avoiding multiple hypotheses, (2) by selecting or interpreting information only to confirm (and not falsify) an established hypothesis, or (3) by not gathering specific information but rather as much information as possible, without considering whether it is diagnostically relevant (Ehrlinger et al., 2016; Wilke and Mata, 2012). Such cognitive processes lead to biased judgments and inaccurate judgments, which can negatively affect the further learning process of the involved students. One aim of teacher training must be to improve the quality of diagnostic judgments and consequently should focus on the perception of diagnostic situations and the selection and processing of information in a more reflective and analytic way.

However, the cognitive processes underlying judgments also depend crucially on the availability and quality of the information to be processed. Therefore, an approach to optimize the diagnostic process seems promising: Some research has shown that low availability or restrictions (e.g., by lacking time, difficult access to students or high effort) of information can lead to augmented attentional allocation and thus increase the quality of information processing (Esterman and Rothlein, 2019). Thus, teachers may make more reflective decisions throughout the judgment process if information is restricted.

The present study addresses the question of whether a restricted availability of information for judgment (in this case, a selection of tasks that can be solved by a student) influences diagnostically inexperienced prospective teachers’ choice of information, observed biases and final judgment accuracy to derive consequences for the training of future teachers. Following the research strategy for studying judgment processes by Loibl et al. (2020), we propose a cognitive model that builds on the process of (social) hypothesis testing (Trope and Liberman, 1996), and we investigate the effects of a restriction of information availability on the assumed judgment processes and cognitive biases on diagnostically inexperienced prospective teachers when diagnosing misconceptions. We therefore systematically vary the availability of information in a complex diagnostic situation and observe the perception of the situation and the processing of information via log-file data. It is assumed that a restriction of information increases the time spent on more relevant information, leads to an adequate processing of information and thus reduces biases.

## 2 Theoretical background

Studies on educational judgment accuracy focus on various fields, such as teachers’ judgments of their students’ academic achievement, of task difficulty, of cognitive abilities, of motivation or of study skills. They have shown very heterogeneous results concerning the accuracy of judgment but also moderators of judgment accuracy (for reviews, see Südkamp et al., 2012 and Urhahne and Wijnia, 2021). These results emphasize that the accuracy of teachers’ judgments regarding the performance of their students should be improved (van de Pol et al., 2021), but the correlational studies provide only limited explanations for the heterogeneous judgment accuracy. A possible explanation for this moderate judgment accuracy can be found in the cognitively demanding diagnostic situation in that teachers are subject to cognitive biases. Research has shown that in complex situations—as judgments of students’ misconceptions—teachers also select irrelevant information and process it to confirm only a possible misconception without looking for its disproof (Rieu et al., 2024). On the basis of the assumption that judgment processes can be modeled as information processing (Loibl et al., 2020), the present study will assess the influence of the restriction of information availability on judgment processes and its accuracy.

### 2.1 (Teachers’) information processing in decision-making

Teachers assess the learning prerequisites of their students on the basis of various information, which they gather in different teaching situations with different instruments, such as tests, diagnostic conversations, observations of the behavior of students or administered tasks. Following the cue utilization approach (Koriat, 1997), teachers form their judgments in processing certain information (i.e., cues) they may have collected about their students or that is available in the diagnostic situation (e.g., students’ solution to a task, characteristics of a task, etc.). The available information may be more or less relevant to diagnosing student thinking (Brunswik, 1956; Kron et al., 2021; Thiede et al., 2010). To perceive the relevance of the cues that are present in a diagnostic situation, teachers refer to their knowledge (e.g., specific pedagogical content knowledge (PCK), Rieu et al., 2022), which influences judgment processes and thereby judgment accuracy (Loibl et al., 2020). For example, when diagnosing students’ existing misconceptions, teachers must process the available information on the basis of their PCK by first assuming possible misconceptions (and excluding others if necessary), identifying relevant information and making a final judgment by comparing their initial hypotheses with further information (Rieu et al., 2024). This example shows how complex diagnostic situations and the associated cognitive processes may be and how much they depend on the amount and relevance of the available information.

Concerning the impact of the information offered by a diagnostic situation on teacher judgments, Oudman et al. (2018) provide first insights: The authors investigated the effects of the availability of cue stimuli about learners on the judgment accuracy of teachers who were asked to predict whether their own students solve decimal fraction tasks correctly or incorrectly. The available information differed in three conditions: teachers received either the names of the learners (and thus may refer to all information about the student stored in memory), the previous solution of a student to a similar task, or both. The highest judgment accuracy for correct solutions was achieved when teachers had at their disposal the names of the students. In summary, teachers achieved the highest judgment accuracy for incorrect answers when they based their judgments only on the learner’s previous solution. This result indicates that the type of processed information (performance cues) influences judgment accuracy, but it does not answer the question of what amount is necessary to form an accurate judgment. This result was further supported by van de Pol et al. (2024), who reported that the availability of diagnostic cues enhanced teachers’ judgment accuracy in terms of students’ text comprehension.

Outside the educational context, studies have examined the extent to which the amount of available information affects judgments. The results from the fields of marketing and health care show that both too little and too much information degrade the quality of information processing and resulting judgments (Kurtzman and Greene, 2016; Sicilia and Ruiz, 2010). For example, the available information on websites affects processing: if the amount of information on the website increases, it offers more opportunities for the consumer to process the information. However, when more information is available than the consumer is able to store, the consumer has a lower capacity to assimilate and elaborate the information (Sicilia and Ruiz, 2010). Sicilia and Ruiz (2010) describe the dependence of judgment quality on the amount of information as an inverted U-curve: only a certain amount of information can be reasonably processed by readers, too little information is not meaningful, and too much information overloads people. It is reasonable to assume that the available information similarly affects information processing in teacher judgments: a certain amount of information must be available for teachers to make accurate judgments; however, only a restricted amount of information can be processed meaningfully by teachers owing to the high demand for working memory (Sweller, 2011).

The few studies that examine the impact of the reduction in information on judgment processes show that when persons have unrestricted access to information, their confidence increases more than their accuracy does, producing a confidence-accuracy gap (Tsai et al., 2008). This result suggests that when information is reduced, teachers may be less confident in their judgments and thus process the offered information in a more reflective and analytic way. This explanation may be supported by the neurocognitive model of attentional allocation, which states that information processing is influenced by arousal, which enables cognitive and attentional resources and determines the proportion of available resources dedicated to a certain task (Esterman and Rothlein, 2019).

In an educational context, Karst and Bonefeld (2020) assume that augmented attentional allocation indicates stronger attribute-based (i.e., reflective) processing. In their study, when assessing students’ mathematics performance in a virtual classroom, an augmented attentional allocation positively influences more accurate judgment. With the log-file data, they operationalized the amount of attention the students received. These data indicate that teachers with high attentional allocation use information about the student more systematically and in a controlled way than do those with low allocation. The amount of available information was the same for each judge. Hence, the study cannot show a clear interdependence between the amount of processed information and the attentional allocation.

### 2.2 Cognitive biases in educational diagnostic situations

Teachers’ diagnosis of students’ dispositions, for example, their misconceptions, is comparable to judgment processes about other people and their characteristics. Research has shown that in some contexts, people avoid information even if it is accessible and potentially relevant to them. Thus, on the one hand, the availability heuristic (Tversky and Kahneman, 1973) – a cognitive bias in which decisions are made on the basis of readily available information – can occur. On the other hand, cognitive biases such as selective perception, conservatism bias, or confirmation bias can cause information avoidance (Ehrlinger et al., 2016; Nickerson, 1998). Confirmation bias is a tendency to selectively search for information or interpret information in such a way that it confirms one’s preconception or hypothesis (Wilke and Mata, 2012). It results from a failure to critically analyze available information and arises primarily when people find themselves in ambiguous situations that can be interpreted as either consistent or inconsistent with the currently favored hypothesis (Nickerson, 1998). In other words, confirmation bias is the gathering and interpretation of information in a unidirectional way to confirm an initial hypothesis even if there is evidence for at least one other possibility. It is reasonable to assume that such a confirmation bias is especially likely when there is either too little information to contradict the initial hypothesis or too much information to process thoroughly (i.e., an inverted U-curve, as in Sicilia and Ruiz, 2010).

Similarly, in complex diagnostic situations, judgment heuristics lead to biased information processing. In the concrete example of diagnosing a misconception, only one possible misconception could be assumed, or only information that confirms (but does not falsify) a hypothesis can be gathered. Consequently, these biased processes can result in inaccurate judgment (Rieu et al., 2024).

On the basis of the theoretical background, we assume for the present study that the restriction of the available information to process for a diagnostic judgment can lead to augmented attentional allocation and stimulate more reflective processing of the given information (Horstmann et al., 2009; Lohse and Johnson, 1996; Reutskaja et al., 2011; Russo and Dosher, 1983; for an overview, see Orquin and Mueller Loose, 2013). Various studies have shown that attentional allocation is an important factor for processing information and for prospective teachers’ judgment accuracy (Böhmer et al., 2017; Karst and Bonefeld, 2020; Südkamp et al., 2008). A restriction of information therefore seems favorable to avoid the availability heuristic and consequently the rejection of relevant and the processing of irrelevant information.

### 2.3 Cognitive processes in complex diagnostic situations

**Figure 1:**
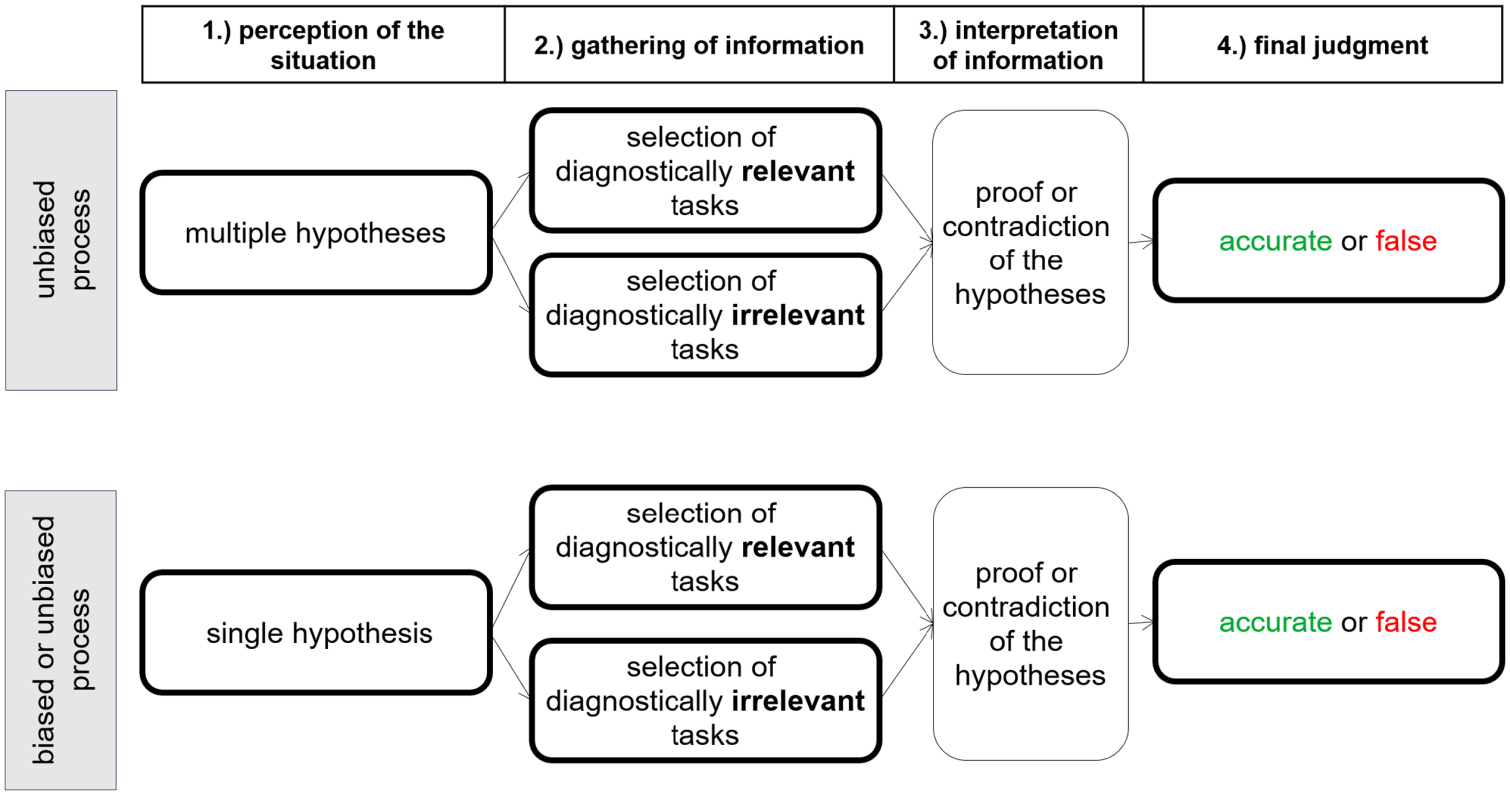
Assumed structure of the judgment process of diagnosing misconceptions in decimal fractions, starting from the formulation of a hypothesis. The bold edges of the boxes represent observable behavior, whereas the fine print edges represent internal processes (according to Rieu et al., 2024).

When the errors of students in solving tasks occur systematically (systematic errors), they can provide evidence of faulty student thinking, so-called misconceptions. In some domains, however, errors cannot be clearly assigned to a single misconception but must be substantiated by further diagnostic steps, such as selecting additional diagnostic tasks. One of these ambiguous and complex judgment situations is the diagnosis of misconceptions in decimal fraction comparisons: different misconceptions may lead to the incorrect solution of a task (Stacey, 2005). Using the situational information of an erroneous student solution and the existing knowledge about possible student dispositions (specific PCK), the teacher can diagnose a possible misconception. Since a task can be solved incorrectly due to several misconceptions, teachers must run a recursive cognitive process to reduce uncertainty and thus make accurate judgments (Rieu et al., 2024). This process can be described ideally in the following steps (see also Figure 1).

First, all possible misconceptions in the situation should be assumed to be causes of the error.

The gathering of further information can be performed via the selection of diagnostically relevant or irrelevant information. In the situation described above, the teacher would ask the learner to solve another task. This task can help distinguish between different misconceptions (diagnostically relevant information) or cannot help distinguishing (diagnostically irrelevant information).

Proving or rejecting hypotheses should take place in a knowledge-based manner on the basis of further information.

The information received now may already be sufficient to validate a hypothesis, i.e., to clearly diagnose one misconception. In the case of uncertainty, the teacher should ask the learner to solve another task to obtain enough information to reject or validate a hypothesis.

The diagnostic situation may offer a large amount of (relevant and irrelevant) information to a teacher in the classroom; thus, the judgment process (Figure 1) can be very demanding for working memory (Sweller, 2011). It therefore seems plausible that teachers, especially prospective teachers without prior knowledge on diagnosing decimal fraction tasks, in this situation are subject to judgment biases such as confirmation bias because they do not recognize the ambiguity of this situation and focus only on one possible misconception. Furthermore, they may selectively choose and process information to maintain an initial hypothesis (confirmation bias, cf. Oswald and Grosjean, 2004).

As mentioned above, the judgment process may be influenced by the amount of available information in a diagnostic situation: too much information leads to a lower capacity to assimilate and elaborate on the information (Sicilia and Ruiz, 2010). In contrast, a restriction of information may lead to focused attention. It has been shown that attentional allocation positively influences teachers’ information processing and judgment accuracy (Böhmer et al., 2017; Karst and Bonefeld, 2020; Südkamp et al., 2008). Indicators for reflective judgment processes are the processing of an increased number of diagnostically relevant tasks on one side and more time spent dealing with the information.

In the described situation of diagnosing misconceptions in decimal fractions, the restriction of further available tasks for judgment and students’ solutions may impact (1) the perceptions of the situation, (2) the gathering of information and (3) the interpretation of information. These processes may result in higher judgment accuracy.

On the basis of the theoretical assumption shown in Figure 1, we predict the following biases in the cognitive processes of prospective teachers without prior knowledge in diagnosing decimal fraction tasks:

#### 2.3.1 Perception of the situation

When diagnosing misconceptions in decimal fractions, a pattern of errors cannot be clearly attributed to one misconception (Padberg and Wartha, 2017; Stacey, 2005). In a concrete situation, an incorrect answer from a student (1.74 is less than 1.312) can be attributed to two different misconceptions, namely, “whole number thinking” (whereby learners regard decimal fractions as two natural numbers separated by a decimal point and solve 74 < 312) or “no decimal point misconception” (whereby students overlook the point in decimal fractions and thus compare 174 < 1312).

The diagnosing participant must recognize this ambiguity and thus generate and verify several hypotheses. Focusing on only one hypothesis can be regarded as a biased judgment process. A restriction of further diagnostic tasks that can be selected takes place after the perception of the situation and should therefore not have any impact on this cognitive step.

#### 2.3.2 Gathering of information

Following an ideal judgment process, teachers gather information by administering further tasks after having perceived the ambiguous diagnostic situation and after having formulated multiple hypotheses. The tasks can differ in their content of information (the number of digits, the number of decimal places, the presence of decimal zeros or trailing zeros). Such tasks are—depending on the available information and the diagnostic context—relevant or irrelevant for the judgment; that is, they can or cannot differentiate between multiple hypotheses on misconceptions. To diagnose the misconception, the provided information from the task must be linked with the established hypotheses. Therefore, the teacher mentally checks which further task has the potential to deliver evidence for or against the initial hypotheses. In the described complex judgment situation, teachers tend to select only tasks that potentially confirm and not falsify their hypothesis (confirmation bias). This bias becomes apparent in the selection of more diagnostically irrelevant rather than relevant tasks.

Restricting the available information during the gathering of information should focus the attention of the judging person on the diagnostic relevance of each piece of information and launch a reflective processing of the given information, leading to the selection of more diagnostically relevant instead of irrelevant tasks.

#### 2.3.3 Interpretation of information

When a teacher has generated multiple hypotheses, the learner’s solution to a diagnostic task can be interpreted as evidence for one of the hypotheses and as contradiction to the other hypotheses. If the teacher only established one single hypothesis, the ambiguity of the diagnostic situation has not yet been perceived. This hypothesis can then either correspond to the present misconception or not: if the hypothesis corresponds to the present misconception, the selected task will support the assumption (Wilke and Mata, 2012). If, on the other hand, the initial single hypothesis does not correspond to the present misconception, the solution of further task(s) may contradict the single hypothesis. In this case, the teacher should ideally discard her/his initial hypothesis and create an alternative hypothesis that better fits the available information. The final judgment can be categorized as correct or false.

However, the effect of a confirmation bias that—in the first step—led the teacher to not perceive the ambiguity of the judgment situation is assumed to lead—in this step, the teacher ignores a learner’s solution that refutes the initial hypothesis or even interprets it as evidence for the initial hypothesis. As argued above, this bias likely occurs when there is too much or not enough information available and when this information is not processed reflectively (Nickerson, 1998).

Restricting the availability of information should focus on attentional allocation and thus help teachers recognize the relevance of the information. This should support them in refuting an initially wrong single hypothesis, and teachers should therefore achieve higher judgment accuracy.

In summary, the present study assumes that the information processing of prospective students without prior knowledge on diagnosing decimal fraction tasks in a demanding diagnostic situation (such as the diagnosis of misconceptions in decimal fractions) may be cognitively biased and impact the gathering and interpretation of information. We therefore investigate whether the restriction of available information remedies these cognitive biases. In the specific judgment situation, the restriction of the availability of further tasks to be solved leads to an augmented allocation of participants’ attention and primes the processing of more relevant tasks. It thus should positively influence the judgment accuracy.

## 3 Research questions and hypotheses

The aim of the present study is to investigate the information processing and cognitive biases of prospective teachers without prior knowledge when diagnosing students’ misconceptions with different amounts of available information. For this purpose, the availability of information to be processed is systematically varied (unrestricted and restricted mode) without changing the diagnostic relevance of the available information.

To make an accurate judgment in a complex and ambiguous diagnostic situation such as the diagnosis of misconceptions with decimal fractions, the judging person needs relevant available information. In this situation, a teacher ideally generates multiple hypotheses and selects suitable tasks to prove or refute each of the hypotheses. Owing to the complexity of the situation, cognitive biases can occur so that a hypothesis is only proven and not refuted, i.e., diagnostically irrelevant tasks (=information) are processed.

### 3.1 Restriction of information

It can therefore be assumed that persons in a restricted mode spend more time searching and interpreting each piece of information; they gather less information but select a higher proportion of relevant tasks. If the participants choose a higher proportion of diagnostically relevant tasks to form their judgment, they are less prone to cognitive biases and should reach a higher judgment accuracy.

Judgment time and the proportion of relevant tasks are therefore indicators of increased attentional focus during information processing in the restricted mode.

In summary, we hypothesize that restricting the availability of information leads to.

H1: an increased time spent per piece of information, which can be seen as an indicator of augmented attentional allocation, and.

H2: the selection and processing of a smaller number but more relevant (in comparison to irrelevant) information.

H3: more accurate judgments.

### 3.2 Biased cognitive processes

On the basis of our assumption and the theoretical background, we expect biases in the perception of the diagnostic situation and in the processing of information. That is, some diagnostically inexperienced prospective teachers may not recognize the ambiguity of the possible misconception that may lead to the erroneously solved task. These individuals are more prone to further biased processing. We therefore hypothesize that.

H4: participants who do not recognize the ambiguity of the situation and whose information processing leads to the retention of the initial hypothesis (bias) select a lower proportion of relevant information and achieve a lower accuracy of judgment (compared with participants who recognize the ambiguity of the situation or change their initial hypothesis during information processing).

### 3.3 Effects of restriction of information on cognitive biases

Nevertheless, for teachers who do not recognize the ambiguity of the situation and whose information processing leads to the retention of the original hypothesis (bias), the restriction of the availability of information should affect information processing and thus lead to a reduction in cognitive biases when gathering information and improve judgment accuracy. We hypothesize that in this subsample,

H5: the restriction of the availability of information leads to increased time spent per piece of information, to the selection of a smaller amount of information, to the selection of a greater proportion of relevant information, and to higher judgment accuracy.

## 4 Method

### 4.1 Sample

Data were collected at two different times with cohorts that were as comparable as possible. An *a priori* power analysis was conducted via G*Power version 3.1.9.7 (Faul et al., 2007) to determine the minimum sample size required to test the study hypothesis. The results indicated that the sample size required to achieve 80% power for detecting a medium effect, at a significance criterion of α = 0.05, was *N* = 71 for paired samples *t*-tests. Thus, the obtained sample size of *N* = 81 is adequate to test the study hypothesis.

Data from all the participating prospective teachers who completed the online questionnaire were included in the analysis. All the participants (84% female) studied mathematics as their main subject to become primary school teachers; their average age was 21.75 years (SD = 1.98), and they mainly attended their 2nd semester of university (SD = 1.15). The data collection took place during a regular course of study.

This sample was deliberately selected to control for possible confounding variables. It can be assumed that the participating students have no subject-specific or subject-didactic knowledge in the area of diagnosing misconceptions in decimal fractions through their studies, as they do not represent a content area at the primary level. The necessary PCK on misconceptions was shown during the entire intervention to ensure the most uniform knowledge base possible.

### 4.2 Design

To test the assumptions mentioned in chapter 3 (i.e., that the restriction of information influences the judgment process and even hampers judgment biases), prospective teachers without prior knowledge on diagnosing decimal fraction tasks were confronted with one erroneously solved task per virtual student. Each virtual student was designed so that their responses would systematically correspond to a common misconception in decimal fractions. The diagnostic goal was to precisely determine this misconception for each student. For this purpose, the participating persons can select further tasks in which the learner to be diagnosed is solved consistently according to misconception. The participants could either select only one task out of four (restricted mode) or an unrestricted number of tasks (unrestricted mode). After seeing the student’s answer(s) to the selected task(s), participants could select a maximum of four times from a further group of four tasks (according to their attributed mode one or an unrestricted number of tasks per group) or proceed to provide the final judgment. Thus, participants in the restriction mode could choose a maximum of four tasks (one of each group of tasks) before diagnosing the misconception. The presented tasks were the same for both conditions (i.e., in each group, two tasks with relevant information and two tasks with irrelevant information). The cases were diagnosed without any time pressure.

**Figure 2:**
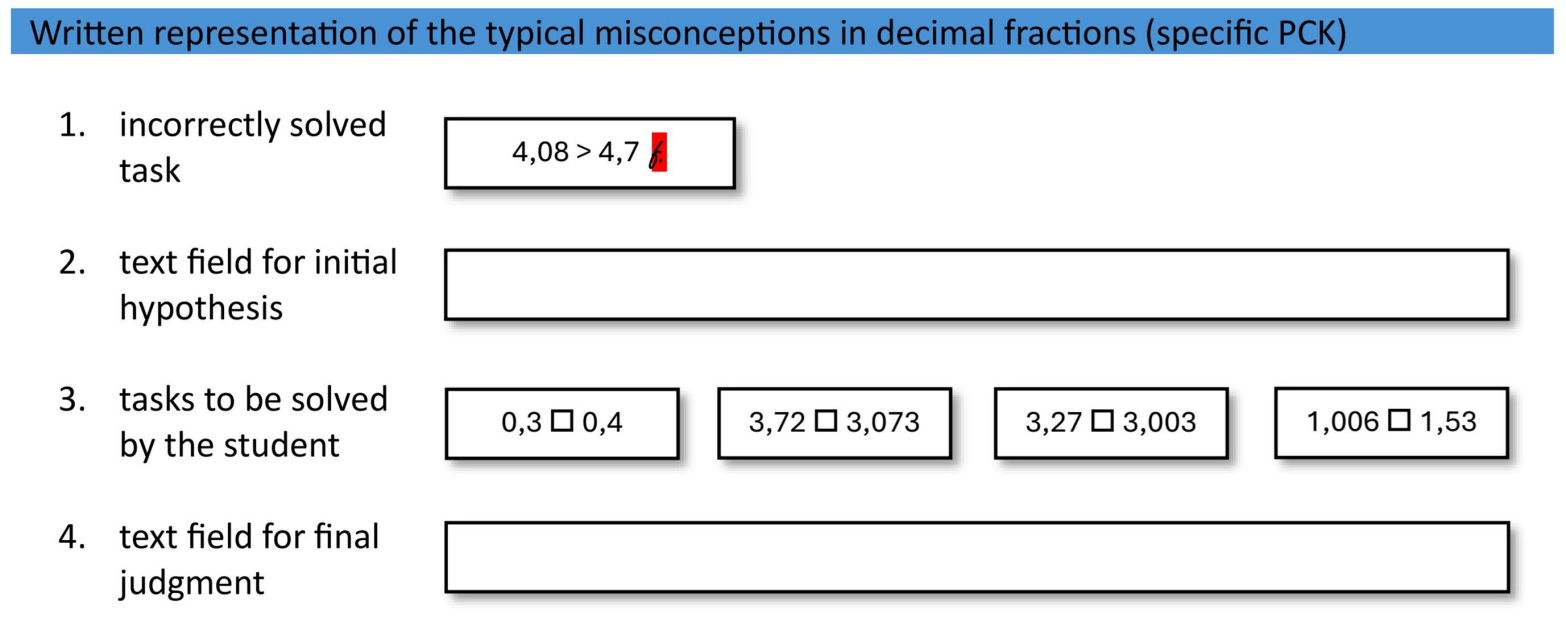
The screen of the element of the diagnostic process was developed for participants, whereas the written representations of the typical misconceptions and the incorrectly solved task were always visible. In the first step, the participants were asked to hypothesize which of the potential misconceptions could be the reason for the presented incorrectly solved task. This formulation was chosen to allow the possibility of writing one or multiple hypotheses.

The participants had to diagnose a total of 5 standardized cases. To control for the influence of specific knowledge facets, we deliberately selected participants without relevant PCK. Therefore, the necessary PCK about the typical misconceptions in decimal fractions was presented before the survey started, and it was always visible to the participants. For each case, the judgment was initiated by a task that had been solved incorrectly by a virtual student (Figure 2). The incorrect solution cannot be clearly attributed to one misconception but represents an ambiguous diagnostic situation in which various misconceptions could have led to the same incorrect solution. In response to the task “Which of the misconceptions does the present student have?,” the prospective teachers first formulate an initial hypothesis about the misconceptions. The participants could enter a single hypothesis or multiple hypotheses.

Afterwards, further tasks that the student to be diagnosed would solve were displayed to the participants. The tasks (presented in groups of four) are classified according to the relevance of their information for the judgment. Relevant information for judgment is found in tasks that provide evidence for a possible misconception in the ambiguous diagnostic situation or contradict another possible misconception. Irrelevant information for judgment is found in tasks that are either solved correctly by all learners despite the presence of any misconception or have the exact same structure as the incorrectly solved tasks presented at the beginning and therefore do not provide any additional information.

To control for other personal characteristics, all test participants were examined in both modes. However, we altered the order of the two modes across participants; that is, some participants started with the restricted mode, whereas others started with the unrestricted mode.

At the end of each case, the participants proceeded to submit the final judgment.

### 4.3 Data collection and scoring

To verify the research assumptions, a digital questionnaire was used to collect (a) the formulated initial hypotheses, (b) the tasks selected and thus processed by the participants, (c) the time spent gathering information and (d) the final judgment.

For (a), we coded the type of hypotheses: single hypothesis or multiple hypotheses. For a single hypothesis, we additionally coded the accuracy of the single hypothesis (i.e., whether the initial hypothesis corresponded to the present misconception) and the congruence with the final judgment (i.e., retention or rejection of the initial hypothesis).

For (b), we counted the number of selected tasks, and we coded the type (diagnostically relevant or irrelevant) of information processed.

For (c), we stopped the time used in the gathering of information and then calculated it on the basis of (b) the time per processed task. A group comparison based on the median split is run to verify the relationship between the time per processed task and the proportion of relevant information.

For (d), we coded the accuracy of the final judgment as correct or false concerning the underlying misconception.

In the first step and corresponding to research hypotheses H1–H3, the influence of the order and availability of restricted and unrestricted information on information processing was examined. Therefore, the collected data about the initial hypothesis, the processed information, the time spent on each task and the final judgment were analyzed and compared with respect to the two modes of availability of information (see chapters 5.1 and 5.2).

In a second step and to verify the assumption that the availability of information plays a role in confirmatory information processing (H4), we compared information processing in judgment processes that can be defined as confirmatory or unbiased (see chapter 5.3). In the ambiguous context of diagnosing misconceptions on the basis of one erroneously solved task, the judging prospective teacher must recognize that at least two misconceptions could have led to the solution and that to diagnose precisely, relevant tasks have to be distinguished from irrelevant tasks that are offered to be solved by the student. Therefore, the initial formulation of multiple hypotheses and the testing of possible options can lead to an accurate diagnosis; this ideal process is considered the normative, unbiased process. After perceiving the ambiguous situation, participants can still select diagnostically irrelevant tasks and even reach incorrect diagnoses without being biased systematically.

Confirmatory biased cognitive processes are initiated by misinterpretation of the diagnostic situation, which leads to the formulation of a single initial hypothesis. This initial hypothesis can match or not match the misconception to be diagnosed. We therefore categorized two cognitive processes as confirmatory biased: the case of a matching single initial hypothesis and its retention (which leads to the correct answer) and the case of a not matching single initial hypothesis and its retention (which leads to an incorrect answer).

In summary, and on the basis of the theoretical background, we define biased judgment processes to be initiated by the formulation of a single hypothesis and to be subject to a tendency to selectively interpret information to confirm one’s hypotheses. Therefore, the processes of confirmatory and unbiased information processing are examined and then substantiated via explorative quantitative analysis of judgment accuracy, the amount and type of processed information and the processing time per task.

## 5 Results

### 5.1 Influence of the order of restricted and unrestricted information on information processing

All the participants engaged in the judgment process with both restricted and unrestricted information. To exclude the effect of individual differences, we first compared the average number of multiple hypotheses, the number and diagnostic relevance of processed information, and the judgment accuracy of the two groups (group 1: first unrestricted mode, second restricted mode vs. group 2: first restricted mode, second unrestricted mode). Bayesian group comparisons were performed via the BayesFactor package (Morey and Rouder, 2022) within R (R Core Team, 2021) to substantiate these results.

We compare the two groups using the Bayes factor, which expresses relative evidence in favor of the null hypothesis (both groups are equal) compared to the alternative hypothesis (there are differences between the groups) and is referred to as BF_01_ (Andraszewicz et al., 2015). The Bayes factors revealed moderate evidence that the two groups are equal concerning formulated hypotheses and the proportion of diagnostically relevant information and anecdotal evidence for equivalence of the two groups regarding the number of processed pieces of information and time per task (see Table 1). Thus, regarding the judgment processes, the two groups do not seem to differ; the order of the two modes can be ignored. However, regarding judgment accuracy, there is anecdotal evidence for differences between the two groups; starting with the unrestricted mode seems to result in a higher overall judgment accuracy. As this is the only difference across groups, we decided to merge the two groups for further analyses but interpret our results regarding judgment accuracy with caution.

**Table 1:** Comparison of the information processed by the two groups.

	Group 1 (*n* = 40): first unrestricted, second restricted mode	Group 2 (*n* = 41): first restricted, second unrestricted mode	
Average number of formulated multiple hypotheses per case (SD)	0.22 (0.37)	0.23 (0.35)	BF_01_ = 5.74
Average number of processed information per case (SD)	3.63 (2.10)	2.93 (1.18)	BF_01_ = 1.24
Proportion of diagnostically relevant information (SD)	0.60 (0.11)	0.57 (0.10)	BF_01_ = 3.10
Judgment accuracy (SD)	0.61 (0.27)	0.48 (0.24)	BF_01_ = 0.61
Average time (in sec.) per processed task (SD)	35.95 (20.46)	43.66 (22.26)	BF_01_ = 1.77

The interpretation of the Bayes factor BF01 as evidence for the null hypothesis follows Andraszewicz et al. (2015).

### 5.2 Influence of the availability of information on the judgment process

The first aim of the study was to show that the availability of information influences the judgment process. In the following, we first examine the assumption that the restriction of the judgment situation changes the type of information that is processed. Therefore, pairwise t-tests with paired groups (restricted and unrestricted mode) were run. Table 2 provides an overview of the average information processed and other judgment parameters contrasting the two modes.

**Table 2:** Overview of the judgment parameters contrasting the two modes.

	Mode	*N* = 81	
Average number of formulated multiple hypotheses per case (SD)	Unrestricted mode Restricted mode	0.23 (0.38) 0.21 (0.36)	*t* (80) = 0.70, *p* = 0.25, *d* = 0.08
Average number of processed information (SD)	Unrestricted mode Restricted mode	5.56 (4.00) 1.27 (0.59)	*t* (80) = 9.83, *p* ≤ 0.001, *d* = 1.09
Proportion of diagnostically relevant information (SD)	Unrestricted mode Restricted mode	0.56 (0.15) 0.68 (0.31)	*t* (80) = −3.15, *p* = 0.001, *d* = −0.35
Judgment accuracy (SD)	Unrestricted mode Restricted mode	0.54 (0.35) 0.54 (0.32)	*t* (80) = 0.18, *p* = 0.43, *d* = 0.20
Average time (in sec.) per processed task (SD)	Unrestricted mode Restricted mode	18.62 (15.70) 65.97 (45.22)	*t* (80) = −9.18, *p* ≤ 0.001, *d* = −1.02

The formulation of the initial hypotheses takes place before information processing and, thus, before the restriction. As expected, the two modes did not influence the average number of formulated multiple hypotheses per case (*t* (80) = 0.70, *p* = 0.25, *d* = 0.08).

As expected (H1), the participants spent significantly more time selecting and processing the information in the restricted mode than in the unrestricted mode (*t* (80) = −9.18, *p* ≤ 0.001, *d* = −1.02). Furthermore, and unsurprisingly, the mode did significantly impact the number of tasks (H2) that the participants processed to make their judgment (*t* (80) = 9.83, *p* ≤ 0.001, *d* = 1.09): When further tasks for the judgment of the misconception were available in the unrestricted mode, a judgment was made using, on average, five further tasks. In the restricted judgment mode, only one or two additional tasks were selected.

The restriction of availability also significantly influenced the type of information selected (*t* (80) = −3.15, *p* = 0.001, *d* = −0.35). As hypothesized (H2), participants selected a greater proportion of diagnostically relevant tasks in the restricted mode than in the unrestricted mode; on average, 68% of all selected tasks were diagnostically relevant. The restriction of available information seems to lead participants to select further tasks more consciously, which could be attributed to the greater attentional focus on the given information. This finding contrasts with the comparison of judgment accuracy. In contrast to our hypothesis (H3), no significant difference between the two modes was found (*t* (80) = 0.18, *p* = 0.43, *d* = 0.20). Despite a higher proportion of diagnostically relevant tasks, participants did not reach more accurate diagnoses in the restricted mode than in the unrestricted mode.

The first hint of a change in information processing due to augmented attentional allocation can be seen in the processing time per task. As seen above, in the restricted mode, the participants spent significantly more time selecting and processing the information than in the unrestricted mode did (see above). A group comparison based on the median split across all case diagnoses (5 cases processed by 81 persons, *N* = 405) shows that augmented attentional allocation can be deduced from the time per task: the more time the diagnosing person spends on tasks, the greater the probability of reflectively selecting diagnostically relevant tasks (*t* (403) = −3.42, *p* = 0.001).

To explain this finding, we will examine the underlying cognitive processes in the next step.

### 5.3 Can the restriction of available information reduce biased cognitive processes?

As stated in H4 and H5, we assume that the availability of information may reduce confirmatory information processing, which is initiated by the formulation of a single initial hypothesis. We therefore examine the extent to which the biased interpretation of the ambiguous judgment situation, which is operationalized by the formulation of a single hypothesis and its retention that leads to the hypothesized judgment, influences the number of processed pieces of information, the proportion of relevant information, the judgment accuracy, and the average time per processed task. For this evaluation, the 5 cases diagnosed from each participant are categorized by the formulation of a single initial vs. multiple initial hypotheses and then examined in a groupwise comparison (see Table 3).

**Table 3:** Descriptive overview of the information processing of judgment processes starting with a single initial hypothesis and its retention until final judgment vs. starting with multiple initial hypotheses or changes in hypotheses in final judgment.

	Biased judgment processes (single initial hypothesis and its retention till final judgment) (*n* = 190)	Normative judgment processes (multiple initial hypotheses or change of hypothesis in final judgment) (*n* = 215)	
Average number of processed information per case (SD)	2.84 (3.18)	3.66 (4.39)	*t* (403) = 2.13, *p* = 0.02, *d* = 0.21
Proportion of diagnostically relevant information (SD)	0.60 (0.37)	0.67 (0.31)	*t* (403) = 2.08, *p* = 0.02, *d* = 0.21
Judgment accuracy (SD)	34.51 (42.25)	44.77 (50.96)	*t* (403) = 2.18, *p* = 0.02, *d* = 0.22
Average time (in sec.) per processed task (SD)	0.44 (0.50)	0.63 (0.48)	*t* (403) = 4.01, *p* < 0.001, *d* = 0.40

According to our assumption (H4), the two types of information processing show exclusively significant differences in the assumed categories: regarding all cases of normative judgment, persons who recognize the ambiguous judgment situation or that change their single initial hypothesis toward another process more and a higher proportion of diagnostically relevant information. They spend more time on each task, and their judgment accuracy is higher.

To answer the question of whether the availability of information influences biased processes, we compare the effects of the restriction of the availability of information on the judgment processes of persons who do not recognize the ambiguous diagnostic situation in a presented case and consequently start with a single initial hypothesis. A total of 189 judgment processes were categorized as biased and differentiated according to the two modes. Table 4 shows the descriptive data of information processing.

**Table 4:** Overview of the influence of restriction of availability on the information processing of biased judgment processes (starting with a single initial hypothesis and its retention until final judgment).

Subgroup of processes starting with a single hypothesis and its retention till final judgment (bias)	unrestricted mode (*n* = 89)	restricted mode (*n* = 100)	
Average number of processed information per case (SD)	4.70 (0.83)	1.20 (0.62)	*t* (188) = 9.05, *p* ≤ 0.001, *d* = 1.32
Proportion of diagnostically relevant information (SD)	0.53 (0.23)	0.66 (0.45)	*t* (188) = −2.44, *p* = 0.01, *d* = −0.36
Average time consumption per task in seconds (SD)	14.03 (15.05)	52.73 (49.76)	*t* (188) = −7.05, *p* ≤ 0.001, *d* = −1.03
Judgment accuracy (SD)	0.43 (0.50)	0.45 (0.50)	*t* (188) = −0.26, *p* = 0.40, *d* = −0.04

In line with our hypothesis (H5), the restriction of availability of information also leads to significant differences for the biased judgment processes (misrecognition of the ambiguous diagnostic situation and retention of the initial hypothesis until the final judgment): in the restricted mode, a smaller number (*t* (188) = 9.05, *p* ≤ 0.001, *d* = 1.32) but a higher ratio of diagnostically relevant information (*t* (188) = −2.44, *p* = 0.01, *d* = −0.36) is processed, and the average time per task increases (*t* (188) = −7.05, *p* ≤ 0.001, *d* = −1.03). The restriction of information has no influence on the judgment accuracy.

## 6 Discussion

The present study addresses the genesis of teachers’ judgments when diagnosing student misconceptions. The main focus was on how the availability of information in the diagnostic situation influences information processing and judgment accuracy. For this purpose, we systematically varied the amount of available information in an experimental design, which was operationalized on the basis of further tasks available to be selected by prospective teachers without prior knowledge, and we observed the processed information.

The participants first saw an erroneous solution to a given task. If the participants recognized that the shown erroneous solution could not be unambiguously attributed to one misconception, they established multiple hypotheses of possible misconceptions and verified them by selecting further tasks and the student’s solutions to these selected tasks. These tasks were either diagnostically relevant or irrelevant; that is, they helped or did not help distinguish between different misconceptions. To calculate judgment accuracy, the participating persons gave their judgment of the misconception. To test the impact of the restriction of information, we systematically varied how many tasks out of a group of four tasks could be selected (unrestricted choice vs. restricted to only one task per presented group of four tasks).

We compared the processing of information during the diagnostic process in the unrestricted and restricted modes. The results show that the possibility of selecting an unrestricted number of further tasks to be solved by a virtual student (information) by prospective teachers led—not surprisingly—to a significantly greater number of selected tasks than when the selection was restricted. Furthermore, the restriction of available information led participants to select a greater proportion of diagnostically relevant tasks, which positively influences judgment accuracy. This result—which could be partly due to the order of the research design (starting with the unrestricted mode helping participants achieve slightly higher judgment accuracy)—is in line with the findings of Oudman et al. (2018) and van de Pol et al. (2024), who showed that the type and availability of processed information influences judgment accuracy and can be attributed to the attentional focus on the relevance of the given information raised in the restricted mode (Esterman and Rothlein, 2019; Karst and Bonefeld, 2020). This augmented attentional focus prompts participants to select the information more consciously—a result that is underlined by the significantly greater amount of time spent per task. The restriction of information leads to a more reflective judgment process, which is characterized by the processing of a higher proportion of diagnostically relevant tasks.

This result can be explained by cognitive biases in the examined judgment processes. People in complex situations do not perceive all the information or process it in a confirmatory way, i.e., in the sense of their first hypothesis (Ehrlinger et al., 2016; Nickerson, 1998). We therefore compared the unbiased and biased judgment processes on the basis of the recognition of the ambiguous diagnostic situation. Unbiased judgment processes start with the formulation of multiple hypotheses, whereas cognitive bias occurs when prospective teachers do not recognize that various misconceptions could be the reason for the incorrectly solved student task and then retain the initial hypothesis until the final judgment. In accordance with our assumption, all examined characteristics of the processing of information show significant differences between biased and unbiased judgments.

To determine the influence of the restriction of available information on cognitive biases, we also compared the restricted and unrestricted modes for biased processes, that is, processes that disregard the ambiguity of the diagnostic situation and then retain the initial single hypothesis until the final judgment. Similarly, for these processes, the results show that less information but a greater proportion of diagnostically relevant tasks were processed during a higher average time in the restricted mode than in the unrestricted mode. Thus, even when prospective teachers do not recognize the ambiguous diagnostic situation and do not generate multiple hypotheses, the restriction of information leads to an augmented attentional allocation (more than three times more time is spent per piece of information) and reduces the biased process. However, given the high proportion of diagnostically relevant tasks and the longer processing time, the restriction of information did not lead to higher judgment accuracy.

In summary, we find similar results for teachers’ information processing compared with other fields: the restriction of available information prompts augmented attentional allocation and reflective information processing, as is apparent in longer processing times and in the selection of a greater proportion of relevant diagnostic information (Böhmer et al., 2017; Karst and Bonefeld, 2020; Orquin and Mueller Loose, 2013; Südkamp et al., 2008). The restriction mode even changes the information processing of persons who do not recognize the ambiguity of the diagnostic situation. It can therefore be concluded that the restriction reduces cognitive biases. Nevertheless, judgment accuracy cannot be improved only by restricting the availability of information. This initially surprising result shows that, in addition to informative framing, intensive training of prospective teachers is also necessary for complex diagnostic processes. One of the central messages in such training curses should be the sensitization to the multicausality of diagnostic situations in the sense of “Look up as many different explanations as possible to the mistakes that the student made here”.

### 6.1 Limitations

We are aware of several limitations in our study and suggest caution in the generalization of the results. Concerning the sample, we examined the diagnostic processes of prospective teachers without prior knowledge and consequently cannot draw conclusions for in-service teachers.

Even if the diagnostic situation described is very realistic and is encountered by teachers in everyday life, the question can still be asked to what extent students without practical experience also perceive this authenticity. Owing to the consistent operationalization of the learners’ misconceptions in the study, the tasks to be selected can be divided into relevant and irrelevant tasks on the basis of their diagnostic potential. In the school context, however, the solution of a task of the same type as the incorrectly solved task at hand (classified as irrelevant information in our study) could represent useful diagnostic information to confirm a misconception.

A cohort of prospective teachers at the beginning of their primary school teaching degree was deliberately selected to reduce the possible influence of different prior subject-specific didactic knowledge. In this way, it can be ensured that the students had very little internship and practical experience and had not yet attended any subject-specific didactic courses as part of their studies. In this way, the experimental nature of the study was taken into account, and supposed disruptive factors were controlled. The prospective teachers had to refer to specific PCK available in written form throughout the study. Therefore, it can be assumed that only predetermined, frequently occurring misconceptions were taken into consideration. Nevertheless, the choice of cohort also represents a limitation to the conclusiveness of the study results: first, the assumption of any individual knowledge was not verified by a survey of prior knowledge; furthermore, only limited statements can be made about teachers in general on the basis of personal characteristics such as the relative diagnostic inexperience of the participants.

The restriction to four frequently occurring misconceptions limits the complexity of the judgment situation, which is not necessarily the case in practice. This restriction is based on the objective of the study and enables the controlled, systematic investigation of the assumed processes.

To exclude the effect of individual differences between participants, we chose a within-subject research design. In contrast to our expectation, we found moderate evidence for differences in judgment accuracy: starting with the unrestricted mode seemed to help participants diagnose a greater number of misconceptions and should be examined in further studies.

Regarding the methodological realization, we deduce that persons who formulated only one single hypothesis did not generate others and thus did not recognize the ambiguity of the diagnostic situation. An additional qualitative follow-up question could provide even more information about the perception of the situation. Since the online survey tool is already very time-consuming and labor-intensive for the participants, we refrained from an additional survey.

The applied procedure of the establishment of a cognitive model and its experimental verification was shown to be an interesting and promising approach for further studies, for example, to test the hypothesis that the amount of information can also be described by an inverted U-curve in the field of teachers’ judgment processes (Sicilia and Ruiz, 2010). These follow-up studies should, for example, address the effects of interventions on the perception of the judgment situation and on the processing of relevant information. On the basis of these results, even clearer statements could be made for teacher training.

## 7 Conclusion

Our study focuses on teachers’ information processing. The findings shed light on the genesis of diagnostic judgments and the influences of cognitive biases. The main result of our study is that the restriction of information leads to the processing of a greater proportion of diagnostically relevant information, even within biased judgment processes. One explanation could be the augmented attentional allocation of each task to diagnose the present misconception. The manipulation of the context (restriction of information) leads to the selection of more relevant information, which positively influences judgment accuracy. The order of the two modes (restriction of information vs. unrestricted access to information) may influence the framing of teacher training. Moreover, the results are highly important for further research on the genesis of teachers’ judgments and, subsequently, for teacher education: an unbiased and accurate judgment process starts with awareness and the perception that diagnostic situations may be ambiguous and characterized by the selection of relevant information. Successful teacher training in the domain of complex diagnostic situations (e.g., misconceptions in decimal fractions) should therefore emphasize the following: (1) the multicausality in diagnostic situations and thus the perception of its ambiguity and (2) the processing of the relevant information.

Our study provides important information about prospective teachers’ diagnostic information processing. Since diagnostic situations are often complex and ambiguous, it can be assumed that diagnostically unexperienced prospective teachers do not perceive all possible sources of error and process irrelevant diagnostic information into judgments. Teacher training should focus more on the perception of diagnostic situations and the deliberate processing of information and thus support teachers in challenging professional activities with regard to adaptive teaching.

## Data Availability

The raw data supporting the conclusions of this article will be made available by the authors, without undue reservation.
