# Supplementary material for: How context influences the processing of relevant information and judgment accuracy—the role of information restriction in judgment processes in diagnosing misconceptions
Source: Front Psychol. 2024 Sep 25;15:1405756. doi: 10.3389/fpsyg.2024.1405756 (PMC11461244; doi:10.3389/fpsyg.2024.1405756)
Supplement: Supplementary file 1 [file Data_Sheet_1.docx]

**Appendix**

The following table shows the descriptive results after the groups were divided and part of the test was performed.

|  |  | Group 1: first restricted mode  (n = 20) | Group 2: first unrestricted mode  (n = 18) |  |
| --- | --- | --- | --- | --- |
| Average number of formulated multiple hypotheses per case (SD) | Restricted  Unrestricted | 0.23 (0.34)  0.33 (0.46) | 0.26 (0.41)  0.11 (0.20) | t (36) = - 0.28, p = 0.779  t (36) = 1.90, p = 0.066 |
| Average number of processed information per case (SD) | Restricted  Unrestricted | 1.25 (0.34)  3.43 (1.37) | 1.31 (0.81)  3.28 (1.71) | t (36) = - 0.33, p = 0.746  t (36) = 0.31, p = 0.758 |
| Proportion of diagnostically relevant information (SD) | Restricted  Unrestricted | 0.78 (0.30)  1.88 (0.65) | 0.94 (0.35)  1.67 (0.86) | t (36) = - 1.61, p = 0.117  t (36) = 0.88, p = 0.383 |
| Judgment accuracy (SD) | Restricted  Unrestricted | 0.43 (0.34)  0.67 (0.36) | 0.87 (0.26)  0.43 (0.22) | t (36) = - 3.61, p = 0.001*  t (36) = 2.45, p = 0.019* |
| Average time (in sec.) per processed task (SD) | Restricted  Unrestricted | 116.83 (83.10)  39.32 (27.29) | 192.75 (126.55)  65.40 (65.13) | t (36) = - 3.51, p = 0.001*  t (36) = 2.73, p = 0.007* |

TABLE

Comparison of the information processed by the two groups and the order of restricted or unrestricted information mode (Group 1: first restricted mode, second unrestricted mode; Group 2: first unrestricted mode, second restricted mode)).

Significant differences were found in terms of judgment accuracy and average processing time. Judgment accuracy was significantly greater in the second part than in the first part for both groups (t(36) = -3.61, p = 0.001 and t(36) = - 2.45, p = 0.019). The group that started with the restricted mode used significantly less time per processed task than did the group that started with the unrestricted mode for both types of mode. Both effects can be attributed to a learning effect. As the main process measures (type of initial hypotheses, number and type of information processed) did not differ across groups, a potential decrease in motivation over time does not seem to have influenced the process. Therefore, both groups are merged for the main analyses.

**Diagnostic vignettes**

The following abbreviations were used for possible misconceptions: whole number thinking (WNT), no decimal point (NDP), and shorter-is-larger (SIL).

| Case 1  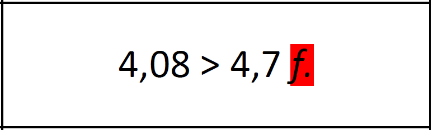  Underlying misconception: WNT  Possible misconceptions: WNT and NDP | *Case 2  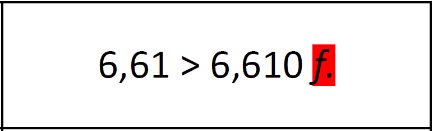  Underlying misconception: SIL  Possible misconceptions: SIL, WNT and NDP |
| --- | --- |
| Case 3  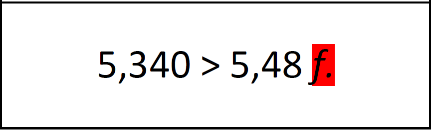  Underlying misconception: NDP  Possible misconceptions: WNT and NDP | Case 4  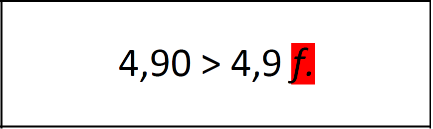  Underlying misconception: NDP  Possible misconceptions: WNT and NDP |
| Case 5  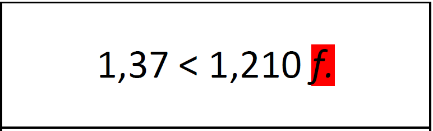  Underlying misconception: WNT  Possible misconceptions: WNT and NDP |  |

** The ambiguity of case 2 is because learners with* WNT and NDP *misconceptions also solve this task incorrectly but swap the inequality sign.*
